# Constructal Equivalent Thermal Resistance Minimization for Tau-Shaped Fin

**DOI:** 10.3390/e22111206

**Published:** 2020-10-25

**Authors:** Shuhuan Wei, Huijun Feng, Lingen Chen, Yanlin Ge

**Affiliations:** 1 Institute of Thermal Science and Power Engineering, Wuhan Institute of Technology, Wuhan 430205, China; weishuhuan2020@163.com (S.W.); huijunfeng@139.com (H.F.); geyali9@hotmail.com (Y.G.); 2 School of Mechanical & Electrical Engineering, Wuhan Institute of Technology, Wuhan 430205, China; 3 College of Power Engineering, Naval University of Engineering, Wuhan 430033, China

**Keywords:** constructal theory, entransy theory, Tau-shaped fin, equivalent thermal resistance, generalized thermodynamic optimization

## Abstract

With the aid of constructal theory and entransy theory, a Tau-shaped fin (TAUSF) is investigated in this paper, and the widths of the bend end and elemental fins are assumed to be different. The construct of the TAUSF is optimized by the minimum equivalent thermal resistance (ETR) obtained by entransy dissipation rate. The constraints of total enveloping volume and fin material volume are considered. The results show that in the specified range of width ratio, the twice minimum ETR of the TAUSF can be yielded by an optimal width ratio and an optimal length ratio. In addition, comparing the optimal performance of the TAUSF with the counterpart of a T-shaped fin, the former sacrifices a small amount of heat transfer performance and its stiffness increases due to its structure with the bend end. The optimal structure of the TAUSF yielded from ETR minimization is conspicuously different with the counterpart yielded from maximum thermal resistance minimization. Comparing the thermal performances of the two optimal constructs, the ETR of the former optimal construct is declined by 10.58%, whereas the maximum thermal resistance is augmented by 5.22%. The former optimal construct can lead to the uniformity of temperature gradient and the reduction in thermal stress, and can guide the engineering designs of practical fins.

## 1. Introduction

Fins are one of the important devices to enhance heat transfer of thermal systems, which is used in electronic device cooling, mechanical equipment cooling, heat exchangers, etc. Based on classical methods, many scholars have implemented many performance analyses of fins by taking entropy generation [1], device temperature [2], heat transfer rate (HTR) [3], and fin efficiency [4] as optimization objectives.

Constructal theory [5,6,7,8,9,10,11,12,13,14,15,16,17,18,19,20,21] has shown its powerful effects in solving various engineering problems, and the constructal optimizations of fins [22,23,24,25,26,27,28,29,30,31,32,33,34] are also one of the foci of this theory. Bejan and Dan [22] first extended the “volume-point” constructal design method in the field of geometry optimization for tree-shaped fins. Bejan and Almogbel [23] performed constructal optimizations of the Tau-shaped fin (TAUSF) and T-shaped fin (TSF) according to the optimization index of maximum HTR. They found that the performance of the TAUSF exhibited a slight decrease compared with that of the TSF, and twice the maximum thermal conductance of the fin could be expressed as a power law of the fin’s parameters. Almogbel [24] investigated the optimal performance of a multi-stage TSF with uniform thicknesses of the stem and branches. Combelles et al. [25] and Chen et al. [26] conducted constructal design for a leaf-like body on the basis of HTR maximization, and improved the HTRs of the leaf-like bodies by optimizing the vein cross-section and thickness of the blade.

Moreover, numerical heat transfer optimization is a tendency for the design of fins, and many researchers have implemented various constructal optimizations of T- [27], Y- [28,29,30,31,32], T-Y- [33], twice Y- [34], and complex-shaped [35] fins by using the finite element method. Almogbel and Bejan [36] performed constructal optimization of pin fins in cylindrical trees. Bello-Ochende et al. [37] investigated pin fins in two rows with specified total fin material volume, and optimized the distribution of fin material. Yang et al. [38,39] and Chen et al. [40] conducted geometry optimizations of cylindrical pin fins in terms of HTR maximization, operation cost minimization, and entropy generation rate minimization, respectively, and obtained different structures of pin fins corresponding to different requirements. Feng et al. [41] and Hajmohammadi [42] further built the helm-shaped and annular fin models, respectively, and improved their performances by using extended surfaces growing from the stem fin. Mustafa [43] carried out constructal design for a diamond-shaped pin fin and gained the optimal spacing and height of the fin under maximum heat transfer density. Hazarika et al. [44] optimized a fork-shaped fin with heat and mass transfer and found that the optimized fin exhibited better HTR than the rectangular fin. 

To consummate the existing heat transfer theory, a new physical quantity, termed as entransy, was brought forward by Guo et al. [45,46]. According to reference [45], entransy was once illuminated as a potential capacity in the heat transfer processes. As stated in entransy theory, an equivalent thermal resistance (ETR) can be defined according to the entransy dissipation rate (EDR) Henceforth, many scholars have applied entransy theory [47,48,49,50] to investigate and improve the heat transfer performances (HTPs) of various thermal systems [51,52,53,54,55,56]. By combining entransy theory and constructal theory, the heat conduction problems [51] as well as convective heat transfer problems of the fins [52,53], heat exchangers [54,56], and vascular networks [55] were solved, and different optimization results and guidelines for the designs of these problems were provided. Moreover, the debate about entransy is a hot topic [57,58,59,60,61,62], whose subjects include the necessity of entransy, physical content of entransy, comparison of entransy and entropy generation, etc. This shows that many constructal optimization results gained by the minimizations of the two performance indexes are different. It is also proved that entransy dissipation is an available optimization objective which provides different design guidelines for the optimizations of various thermal systems. 

Therefore, based on the model established in reference [23], constructal optimization of a TAUSF will be reconducted by utilizing ETR minimization as a performance index in this paper. Furthermore, the widths of the bend end and elemental fins will be assumed to be different, and the performance comparisons of the TAUSF yielded from different performance indexes will be analyzed. The novelty of this paper is introducing entransy theory into the structure optimization of the TAUSF, aimed to increase the uniformity of its temperature gradient. The results obtained here will offer some new references to the developments and HTP improvements of practical fins.

## 2. Model of the TAUSF

**Figure 1:**
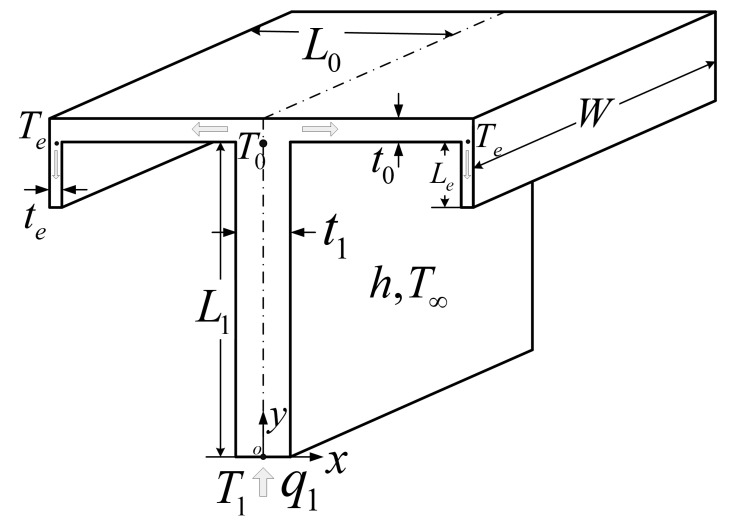
Model of Tau-shaped fin [23].

A model of a TAUSF is depicted in Figure 1 [23]. As shown in Figure 1, the fin consists of one first order fin (width $t_{1}$, length $L_{1}$) and two elemental fins (width $t_{0}$, length $L_{0}$) with a bend end (width $t_{e}$, length $L_{e}$) at the tip of each elemental fin. The structure of the TAUSF is more complex than that of the TSF; thus, its performance is expected to be better for the purpose of electronic device cooling. When the thickness W of the TAUSF is greatly bigger than the lengths $L_{1}$, $L_{0}$, and $L_{e}$, the model of TAUSF can be simplified into a two-dimensional one. The heat current (HTR $q_{1}$, temperature $T_{1}$) generated by the electronic device is imposed at the root of the TAUSF, and then, flows along the first order, elemental, and bend end fins, respectively. The whole heat transfer process is assumed to be steady state. The temperatures at the junctions of the first order and elemental fins and the bend end and elemental fins are $T_{0}$ and $T_{e}$, respectively. The thermal conductivity of the isotropic fin material is k. The tip at the bend end is considered as adiabatic. Natural convection occurs between the TAUSF and air, and the heat transfer coefficient h is uniformly distributed for simplification. The Biot number ${\mathrm{Bi}}_{i}={\left(ht_{i}/k\right)}^{1/2}\;(i=e,0,1)$ of the TAUSF is assumed to be much smaller than 1, and the heat transfer directions in the first order, elemental, and bend end fins can be viewed as one-dimensional ones. The HTR $q_{1}$ and ambient temperature $T_{\infty }$ are specified, and the temperatures $T_{1}$, $T_{0}$, and $T_{e}$ are varied with the shape of the TAUSF. 

For the specified thickness W of the TAUSF, the total enveloping volume and fin material volume are simplified into those of the frontal area A and fin cross-sectional area $A_{f}$, respectively.

(1) $$A=2L_{0}L_{1}$$

(2) $$A_{f}=2L_{e}t_{e}+2L_{0}t_{0}+L_{1}t_{1}$$

For the constructal optimization of the TAUSF, the unchanged A and $A_{f}$ are considered, which means that the fin design is conducted under the conditions of limited space and material consumption. Under these constraints, the optimal structure of the TAUSF is searched to enhance the HTP, which is different from the other design methods aimed to reduce material consumption. The length ratio and width ratio of the fin are defined as: $b_{1}=L_{e}/L_{1}$ and $b_{2}=t_{e}/t_{0}$, respectively, and Equation (2) can be rewritten as

(3) $$A_{f}=2b_{1}b_{2}L_{1}t_{0}+2L_{0}t_{0}+L_{1}t_{1}$$

Equations (1) and (3) can be further nondimensionalized as

(4) $$2{\tilde{L}}_{0}{\tilde{L}}_{1}=1$$

(5) $$2b_{1}b_{2}{\tilde{L}}_{1}{\tilde{t}}_{0}+2{\tilde{L}}_{0}{\tilde{t}}_{0}+{\tilde{L}}_{1}{\tilde{t}}_{1}=\phi_{1}$$

where $({\tilde{t}}_{0},{\tilde{L}}_{0},{\tilde{t}}_{1},{\tilde{L}}_{1})=(t_{0},L_{0},t_{1},L_{1})/A^{1/2}$, and the volume faction of the TAUSF is defined as: $\phi_{1}=A_{f}/A$. From Equations (4) and (5), the dimensionless widths and lengths of the TAUSF can be given as

(6) $${\tilde{t}}_{0}=\frac{{\left(2L_{1}/L_{0}\right)}^{1/2}\phi_{1}}{2+2b_{1}b_{2}(L_{1}/L_{0})+(L_{1}/L_{0})(t_{1}/t_{0})}$$

(7) $${\tilde{t}}_{1}=\frac{{\left(2L_{1}/L_{0}\right)}^{1/2}(t_{1}/t_{0})\phi_{1}}{2+2b_{1}b_{2}(L_{1}/L_{0})+(L_{1}/L_{0})(t_{1}/t_{0})}$$

(8) $${\tilde{L}}_{0}=\frac{1}{{\left(2L_{1}/L_{0}\right)}^{1/2}}$$

(9) $${\tilde{L}}_{1}=\frac{{\left(L_{1}/L_{0}\right)}^{1/2}}{2^{1/2}}$$

The heat transfer equation and boundary conditions of the first order fin are [63]

(10) $$\frac{d^{2}T}{dy^{2}}=\frac{2h}{kt_{1}}(T-T_{\infty })$$

(11) $$y=0,T=T_{1}$$

(12) $$y=L_{1},T=T_{0}$$

Solving Equations (10)–(12) yields the temperature distribution along the first order fin [23].

(13) $$T(y)-T_{\infty }=\csc h(m_{1}L_{1})[(T_{0}-T_{\infty })\sinh(m_{1}y)-(T_{1}-T_{\infty })\sinh(m_{1}y-m_{1}L_{1})]$$

where $m_{1}={\left(\frac{2h}{kt_{1}}\right)}^{1/2}$.

According to reference [38], one can obtain the EDR ${\dot{E}}_{vh\phi }$ of the solid part fin as

(14) $${\dot{E}}_{vh\phi }=\int_{v}k{\left(\nabla T\right)}^{2}dv$$

where v is the volume of the fin and ∇T denotes the temperature gradient. For a one-dimensional problem with a fixed heat flux boundary condition, the optimizations on the basis of the minimizations of EDR and maximum temperature difference are identical to each other [53]. If the optimization based on the latter objective has been conducted, it is meaningless to further conduct the optimization of the TAUSF based on the objective of the whole EDR. Actually, the EDR of the solid part of the TAUSF defined in Equation (14) reflects the uniformity of the temperature gradient, and it is meaningful to conduct the optimization of the TAUSF based on the EDR of the solid part. Based on this consideration, the EDR of the solid part is only considered in the following research unless there are special explanations given. 

From Equation (14), the ETR $R_{h}$ under the specified heat flux boundary condition can be further calculated by [45]

(15) $$R_{h}=\frac{{\dot{E}}_{vh\phi }}{{\dot{Q}}_{h}^{2}}=\frac{\int_{v}k{\left(\nabla T\right)}^{2}dv}{{\dot{Q}}_{h}^{2}}$$

where ${\dot{Q}}_{h}$ denotes the heat current. From Equation (15), for the specified heat current, the EDR minimization is the same as that the ETR minimization in this regard. 

According to Equations (13) and (14), one can obtain the ${\dot{E}}_{vh\phi 1}$ of the first order TAUSF

(16) $$\begin{array}{l} {\dot{E}}_{vh\phi 1}=\int_{0}^{L_{1}}kWt_{1}{\left(\frac{dT}{dy}\right)}^{2}dy=\frac{akW}{4}\csc h(a{\tilde{L}}_{1}{\tilde{t}}_{1}{}^{-1/2}{)}^{2}\{-4a{\tilde{L}}_{1}(T_{0}-T_{\infty })(T_{1}-T_{\infty })\cos h(a{\tilde{L}}_{1}{\tilde{t}}_{1}{}^{-1/2})\\ \;\;\;\;\;\;\;-4{\tilde{t}}_{1}{}^{1/2}\sinh(a{\tilde{L}}_{1}{\tilde{t}}_{1}{}^{-1/2})(T_{0}-T_{\infty })(T_{1}-T_{\infty })+[{\left(T_{0}-T_{\infty }\right)}^{2}+{\left(T_{1}-T_{\infty }\right)}^{2}][2a{\tilde{L}}_{1}+{\tilde{t}}_{1}{}^{1/2}\sinh(2a{\tilde{L}}_{1}{\tilde{t}}_{1}{}^{-1/2})]\} \end{array}$$

where $a={\left(\frac{2hA^{1/2}}{k}\right)}^{1/2}$.

The heat transfer boundary conditions of the elemental fin are similar to those of the first order fin. Following the steps as shown in Equations (10)–(16), the temperature distribution can be derived by

(17) $$T(x)-T_{\infty }=\csc h(m_{0}L_{0})[(T_{e}-T_{\infty })\sinh(m_{0}x)-(T_{0}-T_{\infty })\sinh(m_{0}x-m_{0}L_{0})]$$

In the meantime, the EDR of the elemental fin can be calculated as

(18) $$\begin{array}{l} {\dot{E}}_{vh\phi 0}=\int_{0}^{L_{0}}kWt_{0}{\left(\frac{dT}{dx}\right)}^{2}dx=\frac{akW}{4}\csc h(a{\tilde{L}}_{0}{\tilde{t}}_{0}{}^{-1/2}{)}^{2}\{-4a{\tilde{L}}_{0}(T_{0}-T_{\infty })(T_{e}-T_{\infty })\cos\mathrm{hh}(a{\tilde{L}}_{0}{\tilde{t}}_{0}{}^{-1/2})\\ \;\;\;\;\;\;\;\;-4{\tilde{t}}_{0}{}^{1/2}\sinh h(a{\tilde{L}}_{0}{\tilde{t}}_{0}{}^{-1/2})(T_{0}-T_{\infty })(T_{e}-T_{\infty })+[{\left(T_{0}-T_{\infty }\right)}^{2}+{\left(T_{e}-T_{\infty }\right)}^{2}][2a{\tilde{L}}_{0}+{\tilde{t}}_{0}{}^{1/2}\sinh h(2a{\tilde{L}}_{0}{\tilde{t}}_{0}{}^{-1/2})]\} \end{array}$$

where $m_{0}={\left(\frac{2h}{kt_{0}}\right)}^{1/2}$.

The heat transfer equation and boundary conditions of the bend end fin are [63]

(19) $$\frac{d^{2}T}{dy^{2}}=\frac{2h}{kt_{e}}(T-T_{\infty })$$

(20) $$y=L_{1},T=T_{e}$$

(21) $$y=L_{1}-L_{e},\frac{dT}{dx}=0$$

From Equations (14) and (19)–(21), the temperature distribution and EDR of the bend end can be, respectively, given by

(22) $$T(y)-T_{\infty }=(T_{e}-T_{\infty })\{\cos h[m_{e}(L_{1}-y)]-\tanh(m_{e}L_{e})\sinh[m_{e}(L_{1}-y)]\}$$

(23) $${\dot{E}}_{vh\phi e}=\int_{L_{1}-L_{e}}^{L_{1}}kWt_{e}{\left(\frac{dT}{dy}\right)}^{2}dy=\frac{1}{4}akW{\left(T_{e}-T_{\infty }\right)}^{2}\mathrm{sech}{\left(a{\tilde{L}}_{e}{\tilde{t}}_{e}{}^{-1/2}\right)}^{2}[-2a{\tilde{L}}_{e}+{\tilde{t}}_{e}{}^{1/2}\sinh(2a{\tilde{L}}_{e}{\tilde{t}}_{e}{}^{-1/2})]$$

where $m_{e}={\left(\frac{2h}{kt_{e}}\right)}^{1/2}$.

The heat current gathered at the extremity of the TAUSF and the heat current equations at the interfaces of the elemental, first order, and bend end fins are, respectively, given by

(24) $$q_{1}=-kt_{1}W{\left(\frac{dT}{dy}\right)}_{y=0}=akW{\tilde{t}}_{1}{}^{1/2}\mathrm{cs}\mathrm{ch}(a{\tilde{L}}_{1}{\tilde{t}}_{1}{}^{-1/2})[-(T_{0}-T_{\infty })+(T_{1}-T_{\infty })\cosh(a{\tilde{L}}_{1}{\tilde{t}}_{1}{}^{-1/2})]$$

(25) $$-kt_{1}W{\left(\frac{dT}{dy}\right)}_{y=L_{1}}=-2kt_{0}W{\left(\frac{dT}{dx}\right)}_{x=0}$$

(26) $$-kt_{0}W{\left(\frac{dT}{dx}\right)}_{x=L_{0}}=kt_{e}W{\left(\frac{dT}{dy}\right)}_{y=L_{1}}$$

From Equations (25) and (26), the temperatures $T_{e}$ and $T_{0}$ at the junctions of the fins can be given as

(27) $$\begin{array}{l} T_{e}-T_{\infty }={\tilde{t}}_{0}{}^{1/2}{\tilde{t}}_{1}{}^{1/2}(T_{1}-T_{\infty })\mathrm{cs}\mathrm{ch}(a{\tilde{L}}_{0}{\tilde{t}}_{0}{}^{-1/2})\mathrm{cs}\mathrm{ch}(a{\tilde{L}}_{1}{\tilde{t}}_{1}{}^{-1/2})/[2{\tilde{t}}_{0}\coth{\left(a{\tilde{L}}_{0}{\tilde{t}}_{0}{}^{-1/2}\right)}^{2}\\ \;\;\;\;\;\;\;\;\;\;\;+{\tilde{t}}_{0}{}^{1/2}{\tilde{t}}_{1}{}^{1/2}\coth(a{\tilde{L}}_{0}{\tilde{t}}_{0}{}^{-1/2})\coth(a{\tilde{L}}_{1}{\tilde{t}}_{1}{}^{-1/2})-2{\tilde{t}}_{0}\mathrm{cs}\mathrm{ch}{\left(a{\tilde{L}}_{0}{\tilde{t}}_{0}{}^{-1/2}\right)}^{2}+2{\tilde{t}}_{0}{}^{1/2}{\tilde{t}}_{e}{}^{1/2}\\ \;\;\;\;\;\;\;\;\;\;\;\times \coth(a{\tilde{L}}_{0}{\tilde{t}}_{0}{}^{-1/2})\tanh(a{\tilde{L}}_{e}{\tilde{t}}_{e}{}^{-1/2})+{\tilde{t}}_{1}{}^{1/2}{\tilde{t}}_{e}{}^{1/2}\coth(a{\tilde{L}}_{1}{\tilde{t}}_{1}{}^{-1/2})\tanh(a{\tilde{L}}_{e}{\tilde{t}}_{e}{}^{-1/2})] \end{array}$$

(28) $$\begin{array}{l} T_{0}-T_{\infty }={\tilde{t}}_{1}{}^{1/2}(T_{1}-T_{\infty })\mathrm{cs}\mathrm{ch}(a{\tilde{L}}_{1}{\tilde{t}}_{1}{}^{-1/2})[{\tilde{t}}_{0}{}^{1/2}\coth(a{\tilde{L}}_{0}{\tilde{t}}_{0}{}^{-1/2})+{\tilde{t}}_{e}{}^{1/2}\tanh(a{\tilde{L}}_{e}{\tilde{t}}_{e}{}^{-1/2})]\\ \;\;\;\;\;\;\;\;\;\;\;\times [2{\tilde{t}}_{0}\coth^{2}(a{\tilde{L}}_{0}{\tilde{t}}_{0}{}^{-1/2})+{\tilde{t}}_{0}{}^{1/2}{\tilde{t}}_{1}{}^{1/2}\coth(a{\tilde{L}}_{0}{\tilde{t}}_{0}{}^{-1/2})\coth(a{\tilde{L}}_{1}{\tilde{t}}_{1}{}^{-1/2})-2{\tilde{t}}_{0}\mathrm{cs}{\mathrm{ch}}^{2}(a{\tilde{L}}_{0}{\tilde{t}}_{0}{}^{-1/2})\\ \;\;\;\;\;\;\;\;\;\;\;+2{\tilde{t}}_{0}{}^{1/2}{\tilde{t}}_{e}{}^{1/2}(a{\tilde{L}}_{0}{\tilde{t}}_{0}{}^{-1/2})\tanh(a{\tilde{L}}_{e}{\tilde{t}}_{e}{}^{-1/2})+{\tilde{t}}_{1}{}^{1/2}{\tilde{t}}_{e}{}^{1/2}\coth(a{\tilde{L}}_{1}{\tilde{t}}_{1}{}^{-1/2})\tanh(a{\tilde{L}}_{e}{\tilde{t}}_{e}{}^{-1/2}){]}^{-1} \end{array}$$

From Equations (15), (16), (18), and (23), the total EDR ${\dot{E}}_{vh\phi }$ is calculated as

(29) $${\dot{E}}_{vh\phi }={\dot{E}}_{vh\phi 1}+2{\dot{E}}_{vh\phi 0}+2{\dot{E}}_{vh\phi e}$$

In the meantime, the corresponding dimensionless ETR ${\tilde{R}}_{h}$ of the TAUSF is nondimensionalized as

(30) $$\begin{array}{l} {\tilde{R}}_{h}=\frac{{\dot{E}}_{vh\phi }\cdot (kW)}{q_{1}{}^{2}}=\sinh^{2}(a{\tilde{L}}_{1}{\tilde{t}}_{1}{}^{-1/2})\{2\mathrm{cs}{\mathrm{ch}}^{2}(a{\tilde{L}}_{0}{\tilde{t}}_{0}{}^{-1/2})\{-4a{\tilde{L}}_{0}(T_{0}-T_{\infty })(T_{e}-T_{\infty })\cosh(a{\tilde{L}}_{0}{\tilde{t}}_{0}{}^{-1/2})\\ \;\;\;\;-4{\tilde{t}}_{0}{}^{1/2}(T_{0}-T_{\infty })\;(T_{e}-T_{\infty })\sinh(a{\tilde{L}}_{0}{\tilde{t}}_{0}{}^{-1/2})+[{\left(T_{0}-T_{\infty }\right)}^{2}+{\left(T_{e}-T_{\infty }\right)}^{2}][{\tilde{t}}_{0}{}^{1/2}\sinh(2a{\tilde{L}}_{0}{\tilde{t}}_{0}{}^{-1/2})\\ \;\;\;+2a{\tilde{L}}_{0}]\}+\mathrm{cs}{\mathrm{ch}}^{2}(a{\tilde{L}}_{1}{\tilde{t}}_{1}{}^{-1/2})\{-4a{\tilde{L}}_{1}(T_{0}-T_{\infty })(T_{1}-T_{\infty })\cosh(a{\tilde{L}}_{1}{\tilde{t}}_{1}{}^{-1/2})-4{\tilde{t}}_{1}{}^{1/2}\sinh(a{\tilde{L}}_{1}{\tilde{t}}_{1}{}^{-1/2})\\ \;\;\;\times (T_{0}-T_{\infty })(T_{1}-T_{\infty })+[{\left(T_{0}-T_{\infty }\right)}^{2}+{\left(T_{1}-T_{\infty }\right)}^{2}]\;[2a{\tilde{L}}_{1}+{\tilde{t}}_{1}{}^{1/2}\sinh(2a{\tilde{L}}_{1}{\tilde{t}}_{1}{}^{-1/2})]\}+2{\left(T_{e}-T_{\infty }\right)}^{2}\\ \;\;\;\times \sec h^{2}(a{\tilde{L}}_{e}{\tilde{t}}_{e}{}^{-1/2})[-2a{\tilde{L}}_{e}+{\tilde{t}}_{e}{}^{1/2}\sinh(2a{\tilde{L}}_{e}{\tilde{t}}_{e}{}^{-1/2})]\}/\{4a{\tilde{t}}_{1}{\left[(T_{0}-T_{\infty })-(T_{1}-T_{\infty })\cosh(a{\tilde{L}}_{1}{\tilde{t}}_{1}{}^{-1/2})\right]}^{2}\} \end{array}$$

Substituting Equations (6)–(9), (27), and (28) into Equation (30) yields the ${\tilde{R}}_{h}$ of the TAUSF, which is a function of the fin material fraction $\phi_{1}$, parameter a, length ratios $b_{1}$ and $L_{1}/L_{0}$ as well as width ratios $b_{2}$ and $t_{1}/t_{0}$. When ${\tilde{R}}_{h}$ of the TAUSF is minimized, the temperature field and thermal stress of the solid fin in engineering are more uniform, and the global heat transfer performance of the TAUSF is improved.

## 3. Constructal Optimization for the TAUSF

For the specified $\phi_{1}$, a, $b_{1}$, and $b_{2}$, one can conduct constructal optimizations for the TAUSF by optimizing the two design variables ($L_{1}/L_{0}$ and $t_{1}/t_{0}$). Numerical calculations are employed to gain the minimum EDR and optimal geometry of the TAUSF.

### 3.1. Optimization Based on Two Design Variables

**Figure 2:**
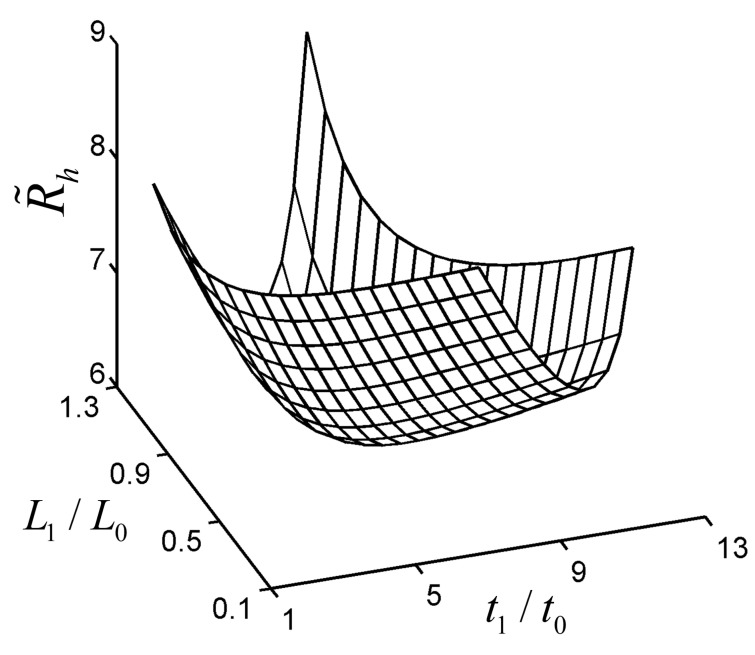
Characteristic of ${\tilde{R}}_{h}$ versus $L_{1}/L_{0}$ and $t_{1}/t_{0}$.

Figure 2 depicts the characteristic of the dimensionless ETR (${\tilde{R}}_{h}$) versus the design variables ($L_{1}/L_{0}$ and $t_{1}/t_{0}$) with $\phi_{1}=0.1$, a=0.2, $b_{1}=0.2$, and $b_{2}=0.2$. From Figure 2, in the ranges of $0.1<L_{1}/L_{0}<1.3$ and $1<t_{1}/t_{0}<13$, there exists an optimal $L_{1}/L_{0}$ (${\left(L_{1}/L_{0}\right)}_{opt}=0.7675$) and an optimal $t_{1}/t_{0}$ (${\left(t_{1}/t_{0}\right)}_{opt}=6.430$), leading to the double minimum ${\tilde{R}}_{h}\cdot$ (${\tilde{R}}_{h,mm}=6.275$) of the TAUSF. 

**Figure 3:**
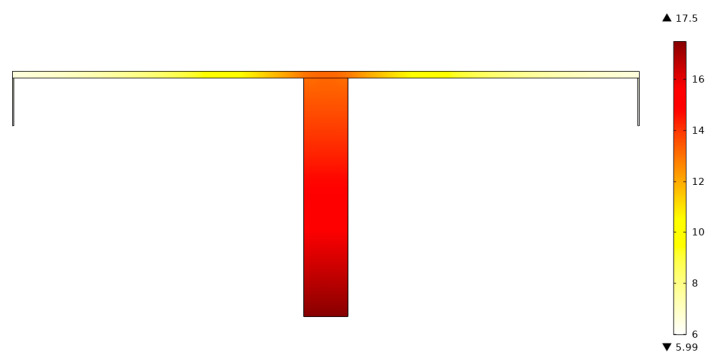
Dimensionless temperature distribution of the Tau-shaped fin (TAUSF) gained by CFD software.

**Figure 4:**
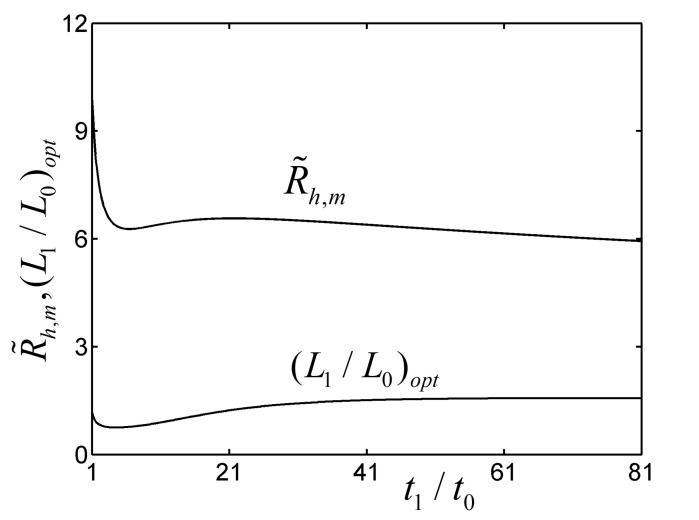
Characteristics of ${\tilde{R}}_{h,m}$ and ${\left(L_{1}/L_{0}\right)}_{opt}$ versus $t_{1}/t_{0}$.

To validate the optimal analytical result of the TAUSF above, the fin model with $L_{1}/L_{0}=0.7675$ and $t_{1}/t_{0}=6.430$ is numerically resolved by computational fluid dynamics (CFD) software (COMSOL Multiphysics 5.0 [64]). It shows that the relative error ($\left|{\tilde{R}}_{h,\;\mathrm{analytical}}-{\tilde{R}}_{h,\;\mathrm{numerical}}\right|/{\tilde{R}}_{h,\;\mathrm{numerical}}$) of the dimensionless ETRs gained by the analytical and numerical solutions is 2.63%, which verifies the correctness of the analytical result in this paper. Furthermore, the dimensionless temperature ($\tilde{T}=(T-T_{\infty })\cdot (kW)/q_{1}$) distribution of the TAUSF gained by CFD software is shown in Figure 3. However, in the ranges of $L_{1}/L_{0}>0$ and $t_{1}/t_{0}>0$, the characteristics in Figure 2 may be invalid, and this case is shown in Figure 4. The minimum ${\tilde{R}}_{h,m}$ is gained by optimizing $L_{1}/L_{0}$, and the characteristic of ${\tilde{R}}_{h,m}$ versus $t_{1}/t_{0}$ is shown in Figure 4. From Figure 4, as $t_{1}/t_{0}$ increases, ${\tilde{R}}_{h,m}$ shows a variation that decreases first, then increases, and at last, decreases. Finally, one can see that the higher the value of $t_{1}/t_{0}$ is, the lower the minimum dimensionless ETR becomes. However, in engineering applications, the width ratio of the fins cannot be infinite, and it is insignificant when $t_{1}/t_{0}$ is large enough. Therefore, the changing range of $t_{1}/t_{0}$ is chosen at $1\leq t_{1}/t_{0}\leq 50$ in this paper, and the double minimum ${\tilde{R}}_{h,mm}$ can be gained under this condition.

**Figure 5:**
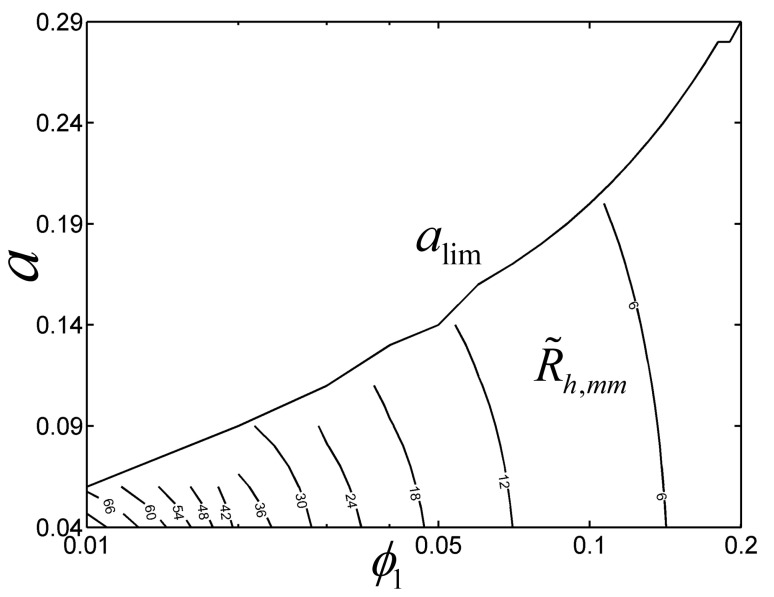
Contour plot of ${\tilde{R}}_{h,mm}$ in the parameter space of $\phi_{1}$ and a.

Within the changing range of $1\leq t_{1}/t_{0}\leq 50$, some values of the parameters $\phi_{1}$ and a cannot satisfy this range. Figure 5 shows the contour plot of the double minimum (${\tilde{R}}_{h,mm}$) in the parameter space of $\phi_{1}$ and a with $b_{1}=0.2$, $b_{2}=0.2$ and $1\leq {\left(t_{1}/t_{0}\right)}_{opt}\leq 50$. From Figure 5, the line $a_{\lim}$ is the critical line which determines whether ${\left(t_{1}/t_{0}\right)}_{opt}$ is lower than 50 or not, and the ${\tilde{R}}_{h}$ of the TAUSF reaches its double minimum ${\tilde{R}}_{h,mm}$ with $1\leq {\left(t_{1}/t_{0}\right)}_{opt}\leq 50$ in the lower part of the critical line $a_{\lim}$. The fitting equation of the critical line $a_{\lim}$ can be given as follows:

(31) $$a_{\lim}=23.821\phi_{1}{}^{3}-10.637\phi_{1}{}^{2}+2.427\phi_{1}+0.0433$$

when $\phi_{1}=0.1$, the critical value of $a_{\lim}$ is 0.2035, and this is why there exists ${\left(L_{1}/L_{0}\right)}_{opt}$ and ${\left(t_{1}/t_{0}\right)}_{opt}\cdot$ (${\left(t_{1}/t_{0}\right)}_{opt}\leq 50$), leading to ${\tilde{R}}_{h,mm}$ in Figure 2.

### 3.2. Parameter Influences on the Optimal Results

**Figure 6:**
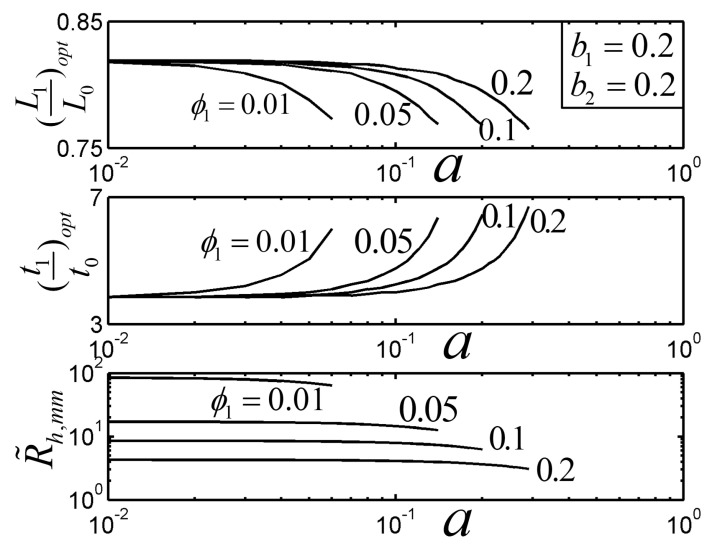
Effect of a on the optimal constructs of the Tau-shaped fin.

Figure 6 depicts the effects of the parameter a on the ${\tilde{R}}_{h,mm}$ of the TAUSF and its corresponding optimal constructs (${\left(L_{1}/L_{0}\right)}_{opt}$ and ${\left(t_{1}/t_{0}\right)}_{opt}$) with $b_{1}=0.2$, $b_{2}=0.2$, and $a\leq a_{\lim}$. From Figure 6, with the increase in a, ${\tilde{R}}_{h,mm}$ decreases, and as a result, the HTP of the TAUSF becomes better. For a small value of a, ${\left(L_{1}/L_{0}\right)}_{opt}$ tends to be 0.8190, and ${\left(t_{1}/t_{0}\right)}_{opt}$ tends to be 3.835; with the increase in a, ${\left(L_{1}/L_{0}\right)}_{opt}$ decreases, ${\left(t_{1}/t_{0}\right)}_{opt}$ increases, the first order fin turns shorter and broader, and the elemental fin becomes smaller. Moreover, the ${\tilde{R}}_{h,mm}$ can be correlated as a function of the parameters a and $\phi_{1}$ within an error of 17.07%

(32) $${\tilde{R}}_{h,mm}=0.58a^{-0.22}\phi_{1}{}^{-0.90}$$

Equation (32) is valid in the ranges of $0.01\leq \phi_{1}\leq 0.2$ and $0.04\leq a\leq a_{\lim}$.

**Figure 7:**
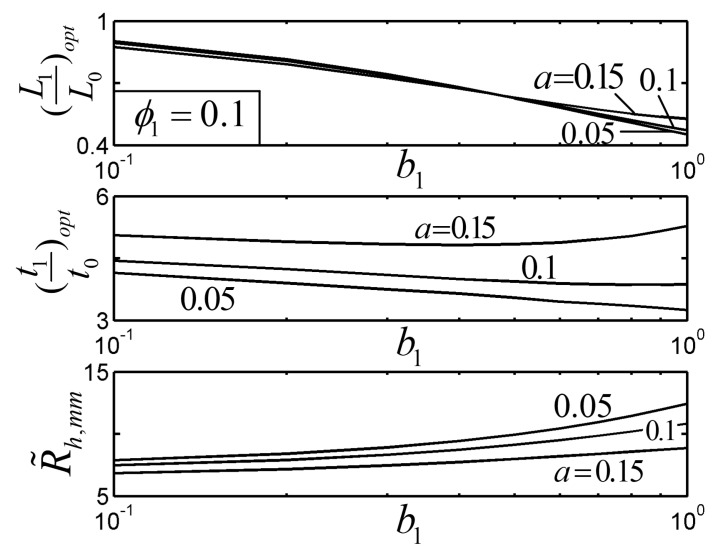
Effect of $b_{1}$ on the optimal constructs of the Tau-shaped fin.

**Figure 8:**
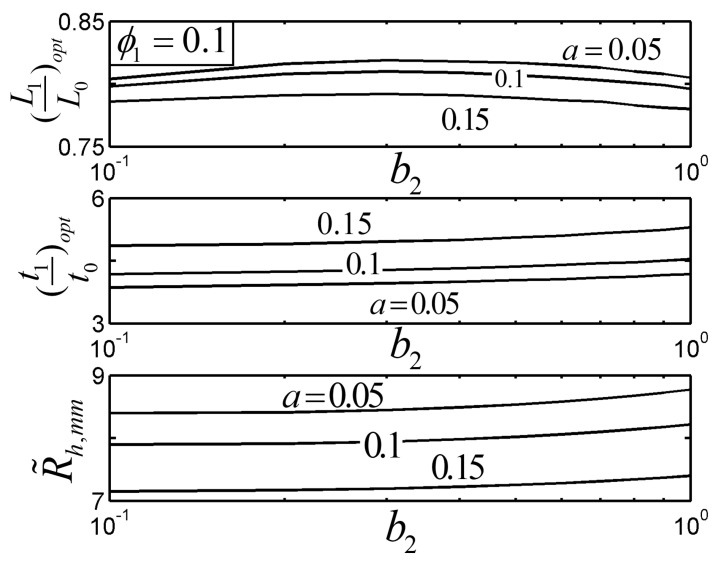
Effect of $b_{2}$ on the optimal constructs of the Tau-shaped fin.

Figure 7 and Figure 8 show the effects of the length ratio and width ratio $b_{2}$ on the ${\tilde{R}}_{h,mm}$ of the TAUSF and its corresponding optimal constructs (${\left(L_{1}/L_{0}\right)}_{opt}$ and ${\left(t_{1}/t_{0}\right)}_{opt}$) with $\phi_{1}=0.1$ and $a\leq a_{\lim}$. From Figure 7, when $b_{1}=1$, the length of the bend end is equal to that of the first order fin, and the HTP of the TAUSF becomes weak; ${\tilde{R}}_{h,mm}$ decreases as $b_{1}$ decreases; the TAUSF with the bend end is simplified into the TSF when $b_{1}=0$. In this case, the HTP of the TAUSF decreases a little compared with that of the TSF, but the stiffness of the TAUSF increases due to its structure with the bend end [23]. From Figure 8, ${\tilde{R}}_{h,mm}$ increases when $b_{2}$ increases; when $b_{2}=1$, the width of the bend end fin is identical to that of the elemental fin. As a result, the HTP of the TAUSF can be improved when different widths ($b_{2}<1$) of the bend end fin and elemental fin are adopted. Moreover, the effect of $b_{2}$ on the optimal geometry of the TAUSF is not obvious than that of $b_{1}$.

### 3.3. Optimal Result Comparison for Different Optimization Objectives

**Figure 9:**
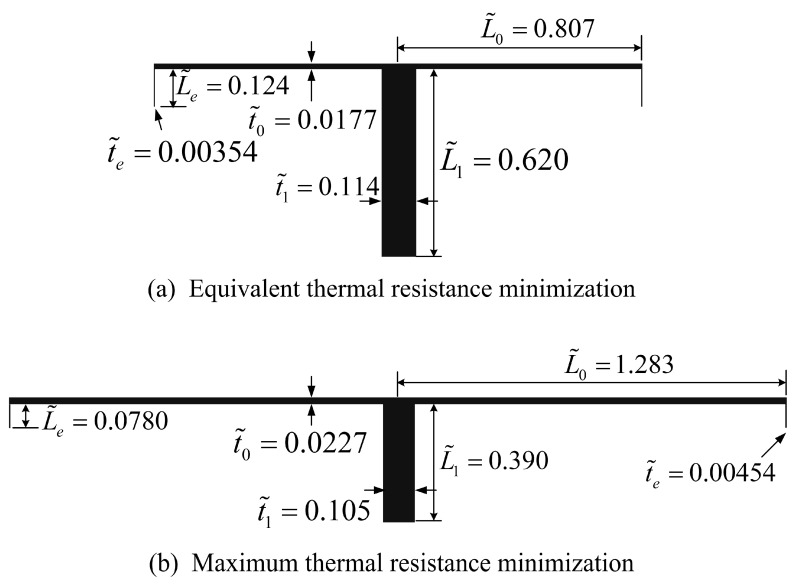
Optimal constructs of the TAUSFs: (**a**) ETR minimization, (**b**) MTR minimization [23].

Furthermore, the optimal geometry of the TAUSF obtained by ETR minimization in this paper is compared with that obtained by maximum thermal resistance (MTR) minimization (its dimensionless form can be defined as: ${\tilde{R}}_{t}=kW(T_{1}-T_{\infty })/q_{1}$, i.e., HTR maximization) in reference [23]. Figure 9 shows the two optimal geometries of the TAUSF with $\phi_{1}=0.1$, a=0.2, $b_{1}=0.2$, and $b_{2}=0.2$. The Biot numbers ${\mathrm{Bi}}_{1}=a{({\tilde{t}}_{1}/2)}^{1/2}$ in Figure 9a,b are 0.0477 and 0.0459, respectively, and the assumption that ${\mathrm{Bi}}_{1}<<1$ is validated. From Figure 9, comparing the optimal geometry of the TAUSF yielded from ETR minimization with the counterpart yielded from MTR minimization, the first order fin grows thicker and longer, the elemental fin becomes thinner and shorter, and the bend end fin becomes shorter and broader. Therefore, the two optimal geometries of the TAUSF are obviously different from each other. For the same A and $A_{f}$ of the TAUSF, the ETR of the TAUSF obtained by ETR minimization decreases by 10.58%, while its MTR increases by 5.22% compared with those obtained by MTR minimization. The optimal geometry of the TAUSF obtained by ETR minimization pursues the minimum average temperature difference of the TAUSF. In this case, the global HTP of the TAUSF is improved, the temperature gradient is more homogenous, and the corresponding thermal stress performance becomes better. The optimal geometry of the TAUSF obtained by MTR minimization pursues the lowest limiting temperature of the TAUSF, which helps to ensure its thermal safety. In practical designs of TAUSFs, the optimal geometry of the TAUSF obtained by ETR minimization can be chosen on the condition that a lower average temperature difference and a homogenous temperature gradient are chased for; that obtained by MTR minimization could be chosen on the conditions that a lower limiting temperature and a higher thermal safety are chased for. Therefore, different optimal geometries of the TAUSFs need to be chosen when different demands of the practical fins should be satisfied. 

## 4. Conclusions

In this paper, the constructal optimization of a TAUSF is re-conducted by utilizing ETR minimization as a performance index according to the model established in reference [23]. The optimal geometry of the TAUSF is obtained. The results show that: 

(1) In the ranges of $0.1<L_{1}/L_{0}<1.3$ and $1<t_{1}/t_{0}<13$, there exists an optimal $L_{1}/L_{0}\cdot ({\left(L_{1}/L_{0}\right)}_{opt}=0.7675)$ and an optimal $t_{1}/t_{0}\cdot$ (${\left(t_{1}/t_{0}\right)}_{opt}=6.430$), leading to the double minimum ETR of the TAUSF. Substituting the optimal $L_{1}/L_{0}$ and $t_{1}/t_{0}$ into Equations (6)–(9) yields the optimal ${\tilde{t}}_{0}$, ${\tilde{L}}_{0}$, ${\tilde{t}}_{1}$, and ${\tilde{L}}_{1}$, and the detailed shape of the TAUSF can be determined for a precise design in engineering. Moreover, the length ratio $t_{1}/t_{0}$ tends to be infinite, and the corresponding ETR can be further decreased.

(2) The HTP of the TAUSF decreases a little in contrast with that of the TSF, but the stiffness of the TAUSF increases due to its structure with the bend end. The corresponding service life and reliability of the TAUSF may be improved from an engineering point of view. In the meantime, the HTP of the TAUSF can be further elevated on the condition that different widths ($b_{2}<1$) of the bend end fin and elemental fin are adopted. 

(3) The optimal geometry of the TAUSF yielded from ETR minimization is different from that yielded from MTR minimization. For the same A and $A_{f}$ of the TAUSF, the ETR of the TAUSF obtained by ETR minimization decreases by 10.58%, and in the meantime, its MTR increases by 5.22%.

Actually, different structures of TAUSFs can satisfy different application demands of the fins. The optimal geometry of the TAUSF obtained by MTR minimization provides a lower limiting temperature and a higher thermal safety for the TAUSF; that based on minimum ETR provides a lower average temperature difference for the TAUSF, and its global HTP is improved. As a result, the optimal geometry of the TAUSF by ETR minimization can offer some new references for the developments and HTP improvements for practical fin designs. The entransy dissipation optimization of the heat conduction problem was experimentally studied in reference [65], but that of the convective problem is only theoretically studied in this paper. Transfer equations about the mechanics are not investigated in this paper, and the stiffness of the fin is only qualitatively described. Therefore, the experimental study of entransy dissipation performance as well as quantitative studies of the stiffness and thermal stress performances will be considered in our future works. 

## Abbreviations

CFD	Computational fluid dynamics
EDR	Entransy dissipation rate
ETR	equivalent thermal resistance
HTR	heat transfer rate
HTP	heat transfer performance
MTR	maximum thermal resistance
TAUSF	Tau-shaped fin
TSF	T-shaped fin

## Nomenclature

*A*	Frontal area, $m^{2}$
$A_{f}$	Fin cross-sectional area, $m^{2}$
a	Parameter related to heat transfer and structure
Bi	Biot number
$b_{1}$	Length ratio
$b_{2}$	Width ratio
${\dot{E}}_{vh\phi }$	Entransy dissipation rate, W·K
*h*	Heat transfer coefficient, $W/m^{2}/K$
k	Thermal conductivity, W/m/K
$L_{0}$	Length of the two elemental fins, m
$L_{1}$	Length of the first order fin, m
$L_{e}$	Length of the bend end, m
$m_{e}$, $m_{1}$	Parameters related to heat transfer and structure
${\dot{Q}}_{h}$	Total heat current, W
$q_{1}$	Heat transfer rate of the heat current, W
$t_{0}$	Width of the two elemental fins, m
$t_{1}$	Width of the first order fin, m
$t_{e}$	Width of the bend end, m
$T_{0}$	Temperature at the junction of the first order and elemental fin, K
$T_{1}$	Inlet temperature of the heat current, K
$T_{e}$	Temperature at the junction of the bend end and elemental fin, K
$T_{\infty }$	Ambient temperature, K
$R_{h}$	Equivalent thermal resistance, K/W
$R_{t}$	Maximum thermal resistance, K/W
v	Volume of the fin, $m^{3}$
W	Thickness, m
x	X axis
y	Y axis
**Greek symbols**	
ϕ	Volume faction of the fin
∇T	Temperature gradient, K/m
**Subscripts**	
*e*	Bend end fin
*lim*	Limited value
*m*	Minimum
*mm*	Double minimum
*opt*	Optimal
0	Elemental fin
1	First order fin
**Superscripts**	
~	Dimensionless
